# Spatial component analysis of MRI data for Alzheimer's disease diagnosis: a Bayesian network approach

**DOI:** 10.3389/fncom.2014.00156

**Published:** 2014-11-26

**Authors:** Ignacio A. Illan, Juan M. Górriz, Javier Ramírez, Anke Meyer-Base

**Affiliations:** ^1^Department of Signal Theory, Networking and Communications, University of GranadaGranada, Spain; ^2^Department of Scientific Computing, Florida State UniversityTallahassee, FL, USA

**Keywords:** Bayesian networks, AD diagnosis, spatial component analysis, magnetic resonance imaging, CAD systems

## Abstract

This work presents a spatial-component (SC) based approach to aid the diagnosis of Alzheimer's disease (AD) using magnetic resonance images. In this approach, the whole brain image is subdivided in regions or spatial components, and a Bayesian network is used to model the dependencies between affected regions of AD. The structure of relations between affected regions allows to detect neurodegeneration with an estimated performance of 88% on more than 400 subjects and predict neurodegeneration with 80% accuracy, supporting the conclusion that modeling the dependencies between components increases the recognition of different patterns of brain degeneration in AD.

## 1. Introduction

Alzheimer's disease (AD) is the most common cause of dementia. The diagnosis of AD is presently based on the presence, in sufficient number, of amyloid plaques and neurofibrillary tangles in cortical brain areas at autopsy. AD has presently no cure, but accurate and non-invasive diagnosis methods are of fundamental importance for increasing benefits from treatments and participate in drug trials.

Since the development of brain imaging techniques, a promising research field has emerged that complements clinical diagnosis in a non-invasive way. Several imaging biomarkers have been studied to characterize AD, as regional cerebral blood flow in SPECT images, and glucose consume or amyloid plaques accumulation in PET imaging. Magnetic resonance imaging (MRI) is a non-invasive technique that provides *in vivo* information about brain structures, encoded in high dimensional data. The structural information of MRI images has been extensively studied for the diagnosis and neurodegeneration prediction of AD, following a vast variety of approaches, including voxel-based morphometry (Chetelat et al., 2002), measures of cortical thickness (Lerch et al., 2008; Eskildsen et al., 2013), or volume measures of specific regions (Davies et al., 2002; Shen et al., 2012). Group comparisons of MRI data, based on statistical techniques, have revealed that several brain regions present deviations from normality in patients with AD, mainly the hippocampus and cortical areas in the temporal, frontal and parietal lobes (Kaye et al., 1997; Killiany et al., 2000). The use of functional MRI (fMRI) imaging for AD has provided further insight into AD characteristics, showing that important relations of connectivity exist between AD affected regions (Kim et al., 2008; Burge et al., 2009). These findings have been proven to be important in the prognosis of AD and the prediction of decline in mild cognitive impairment (MCI) subjects (Greicius et al., 2004). With the advance of machine learning, computer aided diagnosis (CAD) systems have been successfully developed to assist in individual AD diagnosis and neurodegeneration prediction. To this aim, automated MRI-based CAD systems using classification of voxel-based segmented tissues have been proposed (Fan et al., 2008; Kloppel et al., 2008), (see Cuingnet et al., 2011 for a comparative study). An inherent limitation of these methods is that information of spatially separated brain regions is considered as independent, and the effect on classification of information dependencies between AD affected regions are not modeled.

From the statistical pattern-recognition perspective, Bayesian networks are powerful tools for inference that allow for representations of complex relations between dependent features (Friedman et al., 1997). Moreover, modeling these dependencies can increase the classification capabilities, if compared to naive Bayes classification (Cheng and Greiner, 2001). These techniques have been applied in the context of fMRI analysis (Zheng and Rajapakse, 2006; Wu et al., 2011), providing further understanding of complex brain activities. If intended for classification, some alternative modeling is required, as Spatial component (SC) analysis (Gorriz et al., 2008; Illan et al., 2011). In SC, the problem of classification is divided into several independent classification problems by parceling the data of a sample into subdivisions or components and then merging the results together with some aggregation technique. The original motivation to factorize brain images into several smaller pieces is the search of the relevant regions for classification. But simultaneously, the small sample size problem in very high dimensional feature spaces may be tackled by means of a feature dimension reduction. And moreover, it translates the information encoded in MRI images into local features specially designed for classification, so that a Bayesian network approach can be used to produce a final classification decision on each subject.

The pattern of the AD affection is complex, usually involving more than a single brain region. This work focus on modeling the dependencies between affected brain regions for effective diagnosis of AD and early manifestations. We try to model them by performing a Bayesian network learning approach upon the results of classification on regions that are found to be affected by AD. With the use of machine learning techniques, classifiers are able to learn the difference between AD and normal controls from the tissue information on regions. Segmentation methods are used to quantify the probability each voxel to belong to a different brain tissue, and this information is agglutinated in regions, as defined by an anatomical atlas. Extra information concerning AD pathology can be obtained by learning the network structure of dependencies between classification results on each component or region. Once the structure is obtained, the network can be used for inference.

## 2. Material and methods

The build up of the proposed CAD system is divided into three stages: (i) Image pre-processing; (ii) Learning; and (iii) Testing. The first stage is designed for feature extraction. The VBM8 software (Ashburner and Friston, 2000) is used to segment the different brain tissues, followed by a normalization procedure. The ICBM atlas (http://www.loni.usc.edu/atlases/Atlas_Detail.php?atlas_id=5) is used to define the subdivision of spatial components according to anatomical labeled areas. Secondly, the tissue probability on each region is used as feature vectors, and the differences between classes are learned by a support vector machine (SVM, Vapnik, 1998). The binary output define the values of the nodes in a Bayesian network, whose structure is learned following a Markov chain Monte Carlo search on a training sample assuming fully observed data and a Bayesian score. Once the topology is fixed, a maximum likelihood algorithm estimates the values of the network parameters, and the network is finally used for inference. The results of this procedure are compared to other standards in machine learning for CAD. Other aggregation techniques are proposed and compared, as majority voting. Finally, the generalization capability of each proposed CAD is estimated and compared, focusing on mild cognitive impairment subjects with a diagnosis of conversion to AD, to evaluate the diagnosis capability of the methods in early stages of the disease.

### 2.1. Dataset

Data used in the preparation of this article was obtained from the (ADNI) database (http://adni.loni.usc.edu/). The ADNI was launched in 2003 by the National Institute on Aging (NIA), the National Institute of Biomedical Imaging and Bioengineering (NIBIB), the Food and Drug Administration (FDA), private pharmaceutical companies and non-profit organizations, as a 60 million, 5-year public-private partnership. The primary goal of ADNI has been to test whether serial MRI, positron emission tomography (PET), other biological markers, and clinical and neuropsychological assessment can be combined to measure the progression of MCI, and early AD. Determining sensitive and specific markers of very early AD progression is intended to aid researchers and clinicians to develop new treatments, as well as reduce the time and cost of clinical trials.

The Principal Investigator of this initiative is Michael W. Weiner, MD, VA Medical Center and University of California, San Francisco. ADNI is the result of efforts of many co-investigators from a broad range of academic institutions and private corporations, and subjects have been recruited from over 50 sites across the U.S. and Canada. The initial goal of ADNI was to recruit 800 adults, ages 55–90, to participate in the research: approximately 200 cognitively normal older individuals to be followed for 3 years, 400 people with MCI to be followed for 3 years and 200 people with early AD to be followed for 2 years. For up-to-date information, see www.adni-info.org.

In this article, only the data from T1-weighted MR images was considered. The participants were separated into two different classes:

- Normal. Control subjects. Clinical Dementia Rating (CDR) of zero. They were non-depressed, non-MCI and non-demented.
- AD. CDR of 0.5 or 1, met NINCDS/ADRDA criteria for probable AD.

Table 1 shows the demographic details of the subjects who compose the dataset used in this work. Information concerning the conversion from MCI to AD is taken from clinical data available from ADNI. Those patients whose clinical diagnosis suffer multiple conversions and reversions are considered as not reliably labeled and discarded from the MCI-converters cohort.

**Table 1:** **Subject Demographics**.

		**Sex M/F**	**Mean Age/Std**.	**Mean MMSE/Std**.
NC	229	157/72	75.81/4.93	29.06/1.08
MCI-c	110	70/40	76.39/6.96	26.68/2.16
AD	188	123/65	75.33/7.17	22.84/2.91

*NC, Normal Controls; MCI-c, Mild Cognitive Impairment-converters; AD, Alzheimer's Disease*.

### 2.2. Image pre-processing and segmentation

The pre-processing of the images starts with the registration step. It is carried out with the diffeomorphic anatomical registration through exponentiated lie algebra (DARTEL, Ashburner, 2007), which belongs to the VBM8 toolbox within the SPM package (http://dbm.neuro.uni-jena.de/vbm/). The precise inter-subject alignment of DARTEL is followed by the segmentation algorithm of SPM (Ashburner and Friston, 2005). The SPM segmentation algorithm models the intensity value distribution of the T1-weighted MRI and takes voxel location into consideration via a tissue probability map. The images are segmented into three different tissues; gray matter (GM), white matter (WM) and cerebro-spinoid fluid (CSF), producing a set of images with different probability values for GM, WM, or CSF at a given voxel and a cubic resolution of 1.5 mm per voxel. Neither smoothing nor dimension reduction was applied.

### 2.3. Spatial components as bayesian network nodes

The information encoded by MRI images are 3D representations of volume *V* ⊂ ℝ^3^. An image factorization consists of a division of the whole brain image into smaller subvolumes or components, in order to perform the classification task over each component. Explicitly, let the brain image database be *N* 3-D intensity arrays **I**_*i*_(*x_j_*), which denote the voxel intensity at the points *x_j_* ϵ *V* for the brain image patient *i* = 1, …, *N*. The voxel positions *x_j_* form a cubic lattice and the intensity distribution is discretized inside *V*, analogous to 2D image pixel sampling. Let the set *C* = {*x_i_*: *x_i_* ϵ C ⊂ *V*} define a subvolume of *V*. If the volume *V* is subdivided into *M* subvolumes *C*_1_, *C*_2_, …, *C_M_*, also the whole brain image **I**_*i*_(*V*) may be decomposed into the same number of subsets or components **I**_*i*_(*C*_1_), **I**_*i*_(*C*_2_), …, **I**_*i*_(*C_M_*)

A labeled template is used to define the coordinates of each anatomical region recognized here as spatial components. The ICBM (International Consortium for Brain Mapping) high-resolution single subject template is used to this aim, which is aligned in the MNI space (Mazziotta et al., 1995). Each component **I**_*i*_(*C_m_*) selects an anatomical region of the brain image defined in the atlas by means of a set of voxels. The voxel intensities contained in that component are concatenated to a vector,

(1) $$x=(I(x_{1}),I(x_{2}),\ldots ,I(x_{S}))\in {\mathbb{R}}^{S}$$

being *S* the total number of voxels in that component. Each vector is labeled with *y* ϵ ±1, being −1 in case the patient is NORMAL, and +1 in case the patient is AD. These labeled vectors are used as feature vectors for growing *M* SVM classifiers. Thus, the classification task is achieved considering each component individually, provided that the SVM ensemble is aggregated to make a final collective decision.

#### 2.3.1. Background on support vector machines

SVM separate a given set of binary labeled training data with a hyperplane that is maximally distant from the two classes (known as the maximal margin hyper-plane). The objective is to build a function *f*: ℝ^*S*^ → {±1} using the training data from (1), consisting of a size *l* subset of *S*-dimensional patterns **x**_*i*_ and class labels *y_i_*:

(2) $$(x_{1},y_{1}),(x_{2},y_{2}),\ldots ,(x_{l},y_{l})\in \left({\mathbb{R}}^{S}\times \{\pm 1\}\right),$$

so that *f* will correctly classify new examples (**x**′, *y*).

Linear discriminant functions define decision hypersurfaces or hyperplanes in a multidimensional feature space, that is:

(3) $$g(x)=w^{T}x+w_{0}=0,$$

where ***w*** is known as the weight vector and *w*_0_ as the threshold. The weight vector ***w*** is orthogonal to the decision hyperplane and the margin is inversely proportional to its norm. Therefore, the optimization task consists of finding the unknown parameters *w_k_*, *k* = 1, …, *S* by minimizing the norm of the vector ***w*** subject to some linear constraints defining the class belongings, that is:

(4) $$\arg\underset{w,w_{0}}{\min}\underset{\alpha \geq 0}{\max}\left\{\frac{1}{2}\|w{\|}^{2}-\sum_{i=1}^{2}\alpha_{i}[y_{i}(w\cdot x_{i}-b)-1]\right\}$$

where α_*i*_ are some Lagrange multipliers in case of linear separable training data **x_i_**. The method can be extended to not separable training data by introducing the soft margin concept (Vapnik, 1998). Once the parameters **w** defining the hyperplane are obtained, the sign of the distance from the test sample to the hyperplane, that is, **sign**(*g*(*x*)), generates the decision rule.

The outcome of this decision function *g* is defined on the *m*-th component of the subject *i*, as:

(5) $$g\left(x_{m}^{\left(i\right)}\right)\equiv z_{m}^{\left(i\right)}\in \pm 1$$

where the feature vector **x**^*(i)*^_*m*_ is defined in (1). Therefore, *M* SVMs must be trained in order to fix the parameters of the classifier *g* on each component. Once the SVM is trained, the set of binary outcomes will serve us to define a new decision function based on Bayesian networks. The result is to be compared with the common approach in SC, where individual votes are pasted together to grow a collective decision function by a pasting-votes technique (Breiman, 1999; Illan et al., 2011):

(6) $$F(z^{\left(i\right)})=\sum_{m=1}^{M}z_{m}^{\left(i\right)}$$

It is an unweighed sum of votes that each component casts, classifying the subject *i* as *normal* if *F*(*z*^(*i*)^) > 0, and as *AD* if *F*(*z*^(*i*)^) < 0. Majority voting is a simple and robust method of aggregation for the problem of SVM ensemble aggregation (Kim et al., 2003).

#### 2.3.2. Bayesian networks

Formally, a Bayesian network for a set of labeled random variables {Z,Y} is a pair *G* and θ. The first component, *G*, is a directed acyclic graph with nodes corresponding to the the random variables. The graph *G* encodes the dependence relations between variables, represented by edges. The second component of the pair, namely θ, represents the set of parameters that quantifies the conditional probability distribution in the network. A Bayesian network *B* defines a unique joint probability distribution over U given by:

(7) $$p(z_{1},\ldots ,z_{M}|y)=\prod_{m=1}^{M}p\left(z_{m}|{\mathrm{Pa}}_{z_{m}},y\right)=\prod_{m=1}^{M}\theta_{z_{m}|{\mathrm{Pa}}_{z_{m}}}$$

where Pa_*z_m_*_ denotes the set of parents of *z_m_* in *B*. When the topology of the graph *G* is unknown, a set of training data can be used to learn the structure. The common approach is to introduce a scoring function that evaluates each network in the space of all possible structures and returns either the best one or a sample of the best models according to this function. In general, an exhaustive search in this space is Np-hard (Chickering, 1996), so other efficient algorithms have been proposed. We used here the Metropolis-Hastings (MH) algorithm, an Monte Carlo Markov Chain (MCMC) algorithm, to search the space of graphs to find the optimal structure of the network, and the Bayesian information criterion as the score function to find the optimal model (Heckerman et al., 1995).

Once the network structure is learned, a maximum a posteriori scheme is used to learn the parameters of the model, assuming that all the variables are fully observed.

When all the parameters are fixed, the network can be used for inference. If used as a classifier, *B* encodes a distribution *P*(*z*_1_, …, *z_M_*, *y*) so that, given a set of features *z*_1_, … *z_M_*, the classifier returns the label *y* that maximizes the posterior probability *P*(*y*|*z*_1_, …, *z_M_*), which is trivially derived from Equation (7) using the definition of conditional probability and the chain rule.

#### 2.3.3. Evaluation

The classification performance of our approach is tested in three steps: training, cross-validation and test. Cross validation is achieved by means of the leave-one-out method, a technique that iteratively holds out a subject for test, while training the classifier with the remaining subjects, so that each subject is left out once. Three parameters that measure the performance of the classification task are: the Accuracy, the Specificity and the Sensitivity. The definitions are:

$$\begin{array}{l} \text{ Accuracy}=\frac{Tp+Tn}{Tp+Tn+Fp+Fn}\\ \text{Sensitivity}=\frac{Tp}{Tp+Fn}\\ \text{Specificity}=\frac{Tn}{Tn+Fp} \end{array}$$

where *Tp*, *Tn*, *Fp*, and *Fn* denote true positives, true negatives, false positives, and false negatives, respectively.

## 3. Results

The performed experiments start with separating the data into train, test and validation sets. MCI patients are excluded from the modeling cohort and all of them are considered for validation. Therefore, a subgroup of approximately one third of the NC and AD subjects is hold out for validation, and the remaining two thirds are used for train and test phase.

All the images where segmented into GM, WM, and CSF tissue maps aligned in the standard MNI space, allowing for voxel to voxel comparisons with the ICBM altas. The ICBM atlas contains information of 48 brain structures, including WM and CSF tissues. Two possible models are then analyzed depending on the tissue information considered for the component definition: WM or GM. CSF is excluded because it is expected to play an unimportant role.

**Figure 1:**
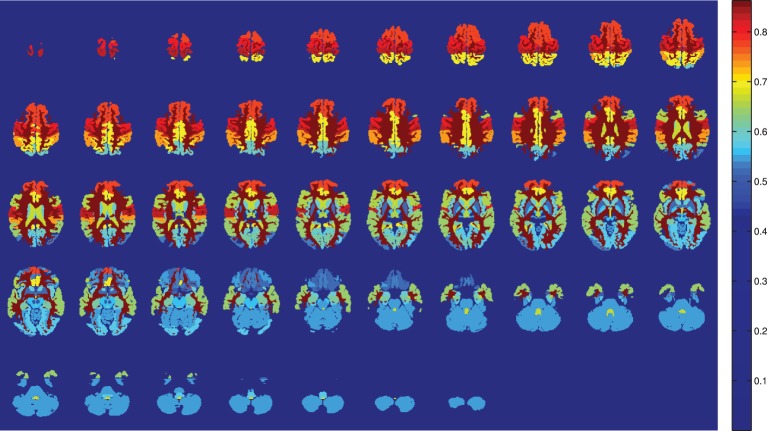
**Training accuracy of the atlas-defined brain regions by the SVM classifiers ensemble**. Axial slices.

**Figure 2:**
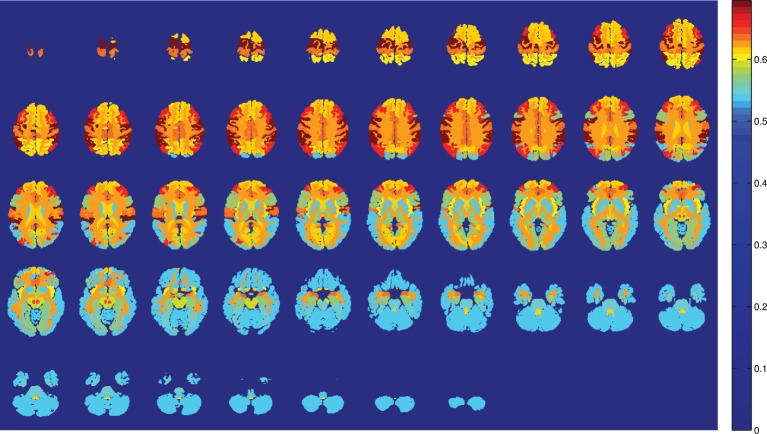
**Training accuracy of the atlas-defined brain regions by the Naive Bayes classifiers ensemble**. Axial slices.

The limited number of available samples determine the cross validation approach in the train-test phase. A leave-one-out cross validation scheme is used to train the classifier of each component, and to estimate its classification performance. Two different classifiers are used to study the classifier influence in the model: SVM and naive Bayes. Figures 1, 2 show the results obtained with SVM classification and Naive Bayes respectively.

**Figure 3:**
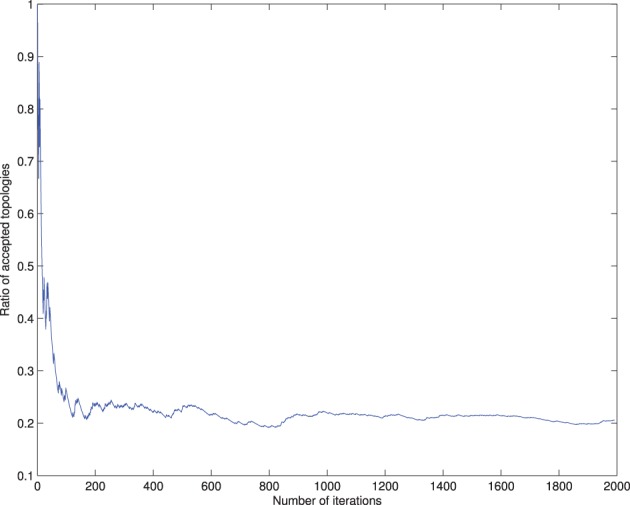
**Convergence of the accepted ratio of the Metropolis-Hasting algorithm for 6 node network**.

**Figure 4:**
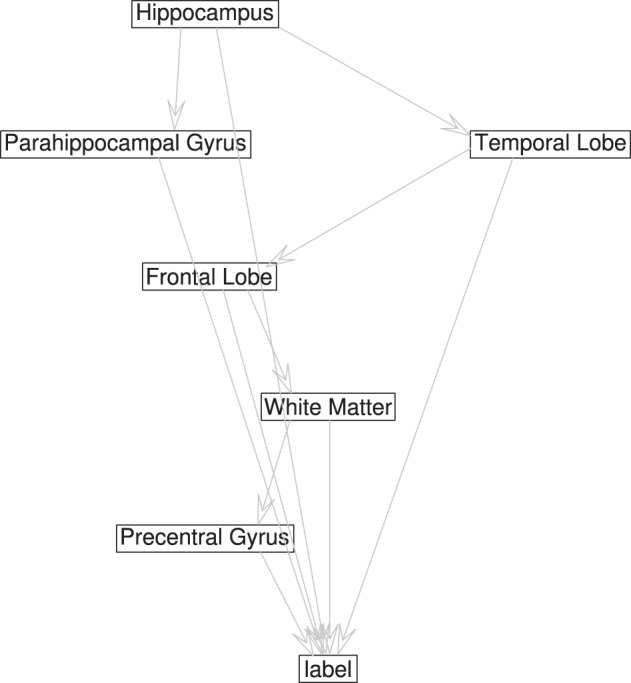
**Topology of the network learned from with maximal BIC score and 6 nodes**.

In the cross-validation step, a 48-dimensional vector of binary values for each image is generated, **z** = {*z*_1_, …, *z*_48_, *y*}, with *z_i_* ϵ ±1 and *y* the class variable. The information contained in the image is reduced to the class assimilated on each component, which serves to learn the Bayesian network structure that relates the dependencies between components. In order to be used for inference, we set the root node of the network as the class node, that is Π_*y*_ = ∅, and each attribute contains the class variable as its parent (see **Figure 4**). If all components are included in the analysis, there are 2^48^ values of the conditional probability table for each image, without considering any relation between components, and the number of possible graphs is super-exponential in the number of nodes. In order to make the calculations affordable, a criteria to reduce the number of interacting components is necessary. A reasonable choice lies in the balance between selecting a significant amount of relevant regions for AD diagnosis while keeping the number of nodes small to make the acceptance ratio of the MCMC algorithm converge (see Figure 3).

**Figure 5:**
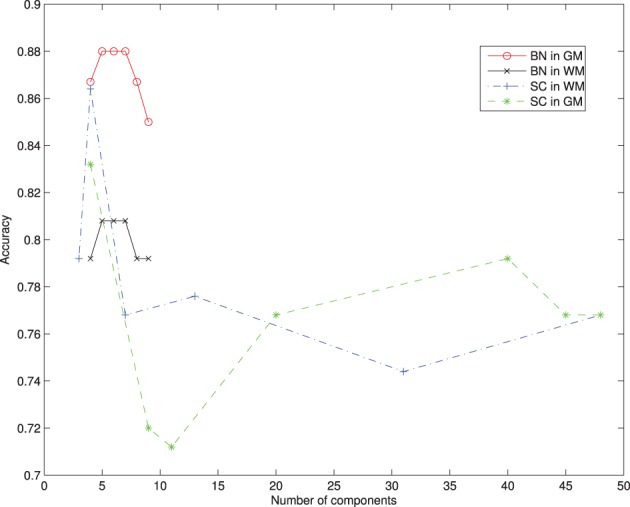
**Recognition rates of AD vs. NC varying the number of components selected from the atlas and comparing the Bayesian Network (BN) approach and the Spatial Component (SC) using voting on different segmented tissues**.

For selecting components, we incorporate the knowledge developed in previous works. The criteria to select components is taken from the literature, and those regions found to play an important role in AD detection are considered, as the parahippocampal gyrus, lingual gyrus, hippocampus, frontal lobe, precentral gyrus, or the temporal lobe (Convit et al., 2000; Killiany et al., 2000; Dickerson et al., 2001; Chetelat et al., 2002). This optimum value for the number of components turns out to be below 10 nodes, in concordance with other works (Wu et al., 2011), guaranteeing convergence. **Figure 5** shows the effect of varying the number of components included to define the network compared to majority voting approach of SC. Figure 3 shows the convergence of the search in the space of all graphs with the Metropolis-Hastings algorithm to a local minimum, and Figure 4 shows the structure obtained for a maximum of the score function. The software package, Bayes Net Toolbox, written by Murphy (2004) was used for structure learning.

The conditional probability distribution (CPD) that fixes the Bayesian-network parameters is learned from the data, provided that some initialization is drawn from a binomial distribution. Informative priors are not required as the data is assumed to be fully observed. In the validation stage, the CPD parameters are used for inference.

The generalization capability of the presented CAD is estimated over the validation set. The validation is performed in two stages, one considering NC vs. AD patients, and other one considering NC vs. MCI-converters. Also, the two possible segmented tissues are analyzed: GM and WM. Performance parameters are calculated, as accuracy, sensibility, specificity and the obtained results are compared to other published methods. The results are summarized in Table 2.

**Table 2:** **Estimated performance parameters for generalization**.

**Set**		**Bayesian Network**		**Voting**	
		**GM%**	**WM%**	**GM%**	**WM%**
AD vs. NC	Accuracy	88.00	80.80	76.80	76.80
	Sensitivity	92.59	81.67	85.92	90.77
	Specificity	84.51	80.00	64.81	61.67
MCI-c vs. NC	Accuracy	80.11	76.57	77.35	75.43
	Sensitivity	77.27	74.55	72.73	72.73
	Specificity	84.51	80.00	84.51	80.00

## 4. Discussion

In this paper we have proposed successful model of dependencies between brain regions for CAD of AD. Remarkably, it shows that previously reported findings of affected brain regions in AD and their relations can be described through a Bayesian network. Considering these relations, the generalization of the CAD system is estimated to be greater than other non-network based approaches, as SC analysis with majority voting aggregation. If compared to other automatic methods using the same database and a comparable number of subjects, the best results obtained with GM tissues are comparable to the best reported ones in NC vs. AD, and superior in the prediction of MCI decline. In Cuingnet et al. (2011), the same database and 162 CN and 137 AD images where used to compare 28 different methods for AD diagnosis. The best reported results from 28 methods reach sensitivity 81% and specificity 95%, comparable to results in Table 2. These results support the idea that the robustness of the automatic diagnosis systems for AD depends on the ability to recognize different presentations of MRI structural anomalies. Moreover, it is straightforward to include in the bayesian network results from classification of regions in other imaging modalities as PIB PET or even neuropsicological tests.

SVM is the optimal classifier if compared to naive Bayes classification, as can be seen from Figures 1, 2. Since the components contain tousends of voxels, a linear kernel SVM can effectively deal with classification problems with low number of samples in high dimensional feature spaces (Vapnik, 1998). Furthermore, GM tissues provide more compelling classification results (see Table 2) as they are expected to capture better the gray matter loss in AD affected patients.

A crucial step in the processing of MRI data is the segmentation of tissues. The framework adopted in this work integrates tissue classification and image registration, as it is required for comparisons with atlases. The model is based on a mixture of Gaussians and is extended to incorporate a smooth intensity variation and nonlinear registration with tissue probability maps (Ashburner, 2007). However, it is expected that AD affected patients will develop some degree of atrophy on determined brain regions. This fact would decrease the segmentation accuracy. This problem is usually solved by providing AD tissue probability maps. From the CAD perspective, introducing class-discriminant knowledge in the processing of the data is not compatible with its goal of classifying new unknown samples. Therefore, some degree of inaccuracy on the segmentation of AD patients is assumed.

The present work is strongly limited by the number of brain regions considered in the Bayesian network and the sample size. It would be desirable to derive the model of dependencies from the whole brain, without introducing any ad-hoc selection, but it has been shown to be impractical. A limitation on the number of brain regions fulfill the requirements to obtain the CAD goal, but some tangential questions may arise. One of them is the question whether a network of dependencies between not registered regions plays an important role in early stages of AD, weakened as the disease progress and translated into the commonly reported ones. Moreover, it might be possible that brain regions that have never been reported to be related to AD, play an important role in the network of dependencies. These questions deserve special attention, and can be important to understand the disease progress. However, in the light of the presented results, it seems unlikely to be the case. From Figure 5, it is deduced that the performance of the bayesian network approach is stable vs. the variation of the component number in contrast with voting approach. It is interesting to stress that the regions involved in the network are not the most accurate, as can be seen in Figure 1. While there is still a margin to improve classification results in the NC vs. MCI-c case, it would be expected that involving more regions in the network would not increase the recognition rates.

The fact that ADNI database is clinically labeled introduces an unavoidable error (Jobst et al., 1998). This error comes from the intrinsic limitations of the clinical assessment, and the generalization of the classifier must be interpreted from this perspective. Taking this fact into account, it can be said that the effective performance of the SC-based CAD denotes an accurate modeling of the dependencies between most relevant brain regions.

## Funding

Data collection and sharing for this project was funded by the Alzheimer's Disease Neuroimaging Initiative (ADNI) (National Institutes of Health Grant U01 AG024904) and DOD ADNI (Department of Defense award number W81XWH-12-20012). ADNI is funded by the National Institute on Aging, the National Institute of Biomedical Imaging and Bioengineering, and through generous contributions from the following: Alzheimers Association; Alzheimers Drug Discovery Foundation; BioClinica, Inc.; Biogen Idec Inc.; Bristol-Myers Squibb Company; Eisai Inc.; Elan Pharmaceuticals, Inc.; Eli Lilly and Company; F. Hoffmann-La Roche Ltd and its affiliated company Genentech, In c.; GE Healthcare; Innogenetics, N.V.; IXICO Ltd.; Janssen Alzheimer Immunotherapy Research & Development, LLC.; Johnson & Johnson Pharmaceutical Research & Development LLC.; Medpace, Inc.; Merck & Co., Inc.; Meso Scale Diagnostics, LLC.; NeuroRx Research; Novartis Pharmaceuticals Corporation; Pfizer Inc.; Piramal Imaging; Servier; Synarc Inc.; and Takeda Pharmaceutical Company. The Canadian Institutes of Health Research is providing funds to support ADNI clinical sites in Canada. Private sector contributions are facilitated by the Foundation for the National Institutes of Health (www.fnih.org).

### Conflict of interest statement

The authors declare that the research was conducted in the absence of any commercial or financial relationships that could be construed as a potential conflict of interest.
